# Gene expression profiling predicts a three-gene expression signature of endometrial adenocarcinoma in a rat model

**DOI:** 10.1186/1475-2867-9-12

**Published:** 2009-05-08

**Authors:** Sandra Karlsson, Björn Olsson, Karin Klinga-Levan

**Affiliations:** 1 Systems Biology Research Centre Biomedicine,, School of Life Sciences, University of Skövde, Box 408, SE-541 28 Skövde, Sweden; 2 Bioinformatics, School of Life Sciences, University of Skövde, Box 408, SE-541 28 Skövde, Sweden

## Abstract

**Background:**

In the Western world, endometrial cancers are the most common gynaecological neoplastic disorders among women. Initial symptoms are often vague and may be confused with several other conditions or disorders. Thus, there is a need for an easy and reliable diagnostic tool. The objective of this work was to identify a gene expression signature specific for endometrial adenocarcinomas to be used for testing potential endometrial biomarkers.

**Results:**

Changes in expression between endometrial adenocarcinomas and non-/pre-malignant endometrium from the BDII EAC rat model were compared in cDNA microarray assays. By employing classification analysis (Weka) on the expression data from approximately 5600 cDNA clones and TDT analysis on genotype data, we identified a three-gene signature (*Gpx3*, *Bgn *and *Tgfb3*). An independent analysis of differential expression, revealed a total of 354 cDNA clones with significant changes in expression. Among the 10 best ranked clones, *Gpx3*, *Bgn *and *Tgfb3 *were found.

**Conclusion:**

Taken together, we present a unique data set of genes with different expression patterns between EACs and non-/pre-malignant endometrium, and specifically we found three genes that were confirmed in two independent analyses. These three genes are candidates for an EAC signature and further evaluations of their involvement in EAC tumorigenesis will be undertaken.

## Background

Endometrial carcinomas (ECs) are the most frequently occurring malignancies in the genital tract among women in the Western world. As in most other cancer diseases, neoplastic progression to EC is very complex, and involves high penetrance genes as well as intricate interactions of multiple low penetrance genes [1]. ECs can be divided into two broad categories based on morphology; Type I endometrial cancer, which accounts for approximately 70–80% of all ECs, follows the estrogen-related pathway and frequently develops in the setting of complex atypical hyperplasias as malignant precursors. The type II category of endometrial tumors is non-endometroid carcinomas which arise from endometrial polyps or from endometrial intra-epithelial carcinoma. Endometrial adenocarcinoma (EAC) is the most common type I EC and originates from the glandular cells of the uterus surface epithelium. Type I tumors occur pre-dominantly in pre- or peri-menopausal women, whereas the more aggressive type II tumors occur in post-menopausal women and carry a high mortality rate [1-3]. Successful treatment is dependent on accurate and early diagnosis. In EAC, however, early presenting symptoms are usually vague and easily confused with other conditions and hence there is a need for an easy and reliable diagnostic tool.

Due to the genetic heterogeneity among the human population and the complexity of tumor etiology, it is attractive to use inbred animal model systems in studies of carcinogenesis. The BDII/Han inbred rat model, first described in 1987, is genetically well characterized and has since then become a useful model for studies of human endometrial adenocarcinoma. More than 90% of the female BDII/Han virgins spontaneously develop EC during their life time, where the majority of the neoplasms are EACs. The endometrial carcinogenesis in the females of the BDII rat strain is hormone dependent and represents an outstanding model for spontaneous hormonal carcinogenesis. [4-6]

Genomic approaches such as gene expression profiling by DNA microarrays, provide unprecedented tools to handle the complexity of cancer at the transcriptional level. In the present study, we have applied different statistical and classification approaches on global expression data to identify potential classifiers, i.e. sets of marker genes whose expression profiles can be used to differentiate between EAC tumors and non/pre-malignant endometrial lesions. Furthermore, we have used genotype data from the female progenies of BDII crosses [7,8] to investigate whether the genes with significantly differential expression are associated to potential susceptibility regions. In this study, a unique dataset has been identified that can act as a starting point to establish a panel of endometrial cancer biomarkers and to explore the role of the identified genes in endometrial carcinogenesis.

## Results

### Significance analysis

**Figure 1:**
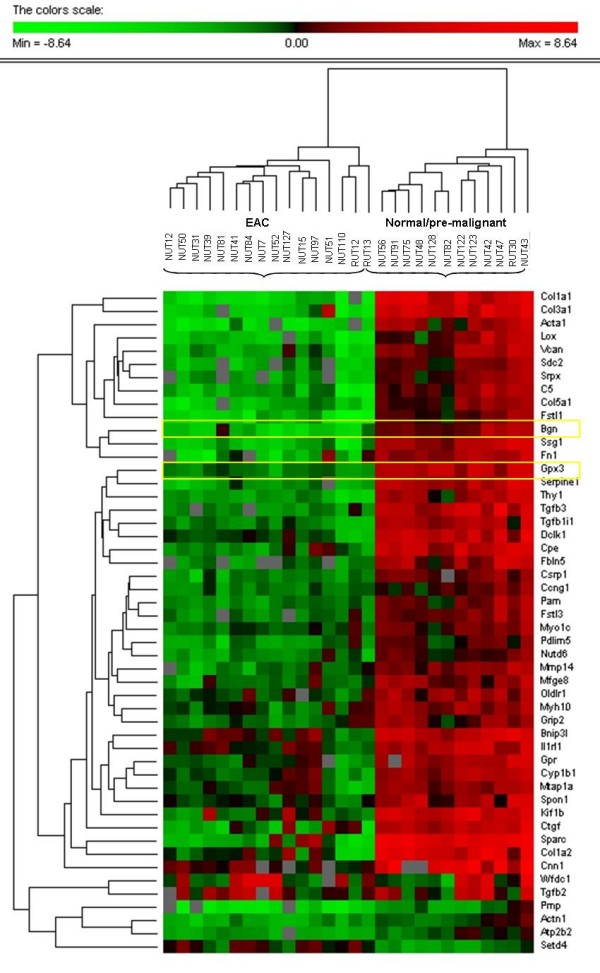
**The results from the hierarchical clustering of the 50 genes with the highest significant differential expression between normal/pre-malignant and EAC samples**. In the significance analysis of the microarray data, 890 genes were found to be differentially expressed between non/pre-malignant samples and the endometrial tumors and when applying FDR, this number was decreased to 354 genes. Here, the 50 genes with the highest differential expression are shown.

The significance analysis of the microarrays demonstrated 890 dysregulated genes. However this number was considerably reduced to 354 when applying FDR *p*-value adjustment. The 50 most differentially expressed genes were subjected to hierarchical clustering and their expressions are shown in Figure 1.

### Gene function classification

**Figure 2:**
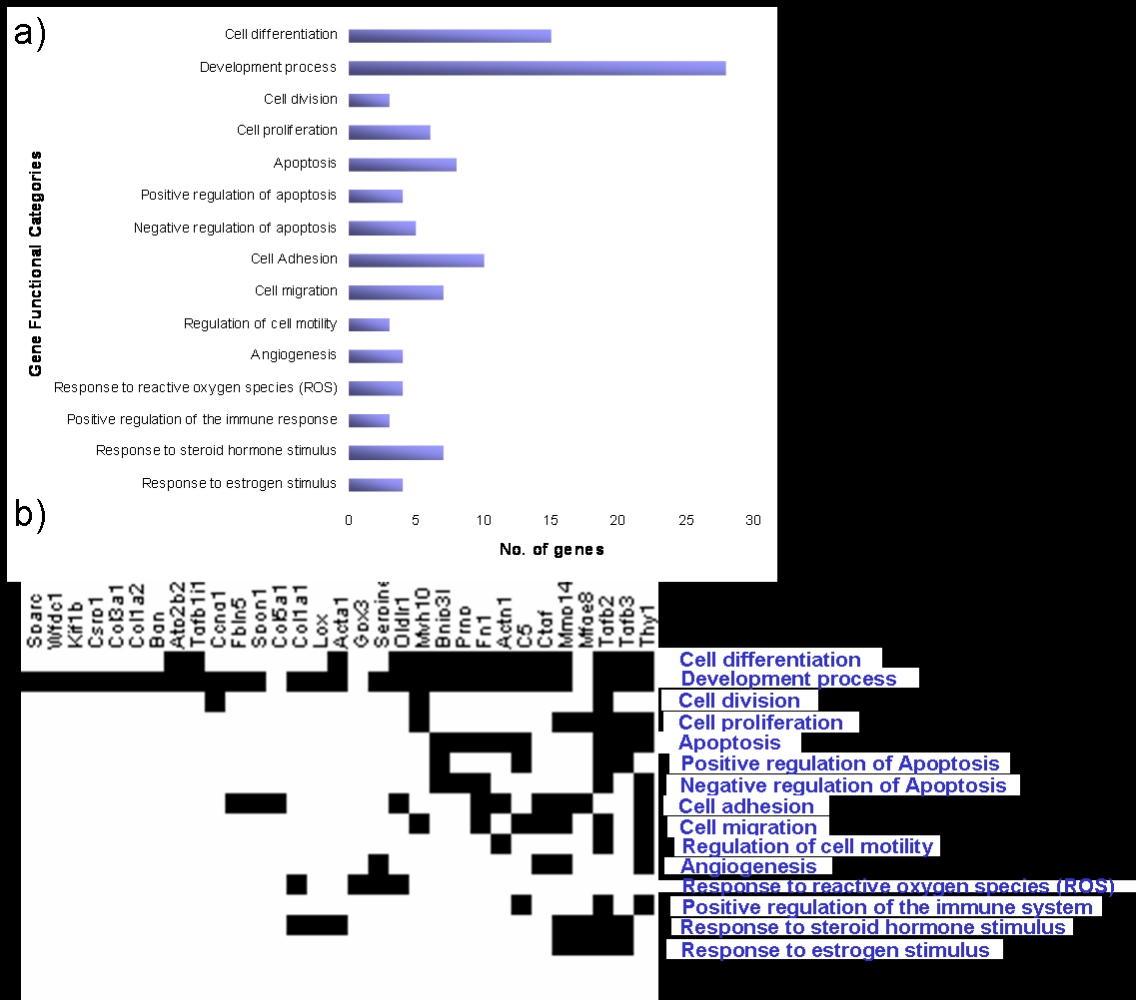
**Gene functional analysis of the 50 genes with the highest significant differential expression**. a) Results of the analysis of gene function revealed that 31 genes were involved in cellular functions frequently implicated in human carcinogenesis, such as apoptosis, proliferation etc. b) Clustering of the functional categories and the 31 identified genes, displayed that several of these genes are involved in several cellular processes.

Out of the top 50 genes, 31 genes were found to be involved in cellular processes commonly implicated in human tumorigenesis (Figure 2a). As illustrated in Figure 2b, a majority of these genes were found to be involved in more than one of these, and in some cases several, processes. For example, *Tgfb2 *is involved in 12 different processes commonly aberrant in tumors and *Thy1 *in 10.

### Identification of gene classifiers

The overall classification results obtained with the models derived by the machine learning algorithms in Weka are summarized in Supplementary Table 1. Each of the 44 applicable algorithms was applied in 29 training and test cycles. In each cycle, a classification was generated using 28 samples as training data and leaving out a single sample for testing (leave-one-out cross validation). The resulting cross-validated average classification accuracy was 97.1%. The algorithm ZeroR serves as a base-line, since it simply classifies every sample as belonging to the majority class, which here results in an accuracy of 59%. All algorithms performed much better than the base-line, with the majority (38 of 43 algorithms) reaching accuracies of 97% (a single misclassification) or 100%.

For many classification algorithms the output from Weka also includes the genes that the classifier is based on, and for some algorithms it is possible to determine the gene that had the most influence on the classification results. Table 1 shows the most frequently occurring of these "top genes", i.e. *Gpx3 *(9 algorithms) and *Bgn *(7 algorithms).

**Table 1:** Potential marker genes most frequently occurring as the best classifiers derived by Weka.

**Ensemble gene Id**	**Gene symbol**	**Gene name**	**Chromosomal localization**
ENSRNOG00000021809	*Gpx3*	Glutathione peroxidase 3	10q22
ENSRNOG00000017440	*Bgn*	Biglycan	Xq37

### TDT analysis

The significance analysis revealed 354 genes to be differentially expressed between non/pre-malignant and endometrial samples. We choose to investigate the locations in the genome of the 50 genes with the highest differential expression. By means of TDT analysis on samples on the backcross progeny (n = 39), we could only identify one gene (*Tgfb3*) with significant association to a susceptibility region (6q31, ~65 cM) in the BN background. In this region, all animals from crosses with the BN background that developed tumors were homozygous for the BDII alleles. We chose to include genes in the proximity (within 10 Mbp or a 20 cM region), which means that there is at least 80% probability that the loci are inherited together.

**Figure 3:**
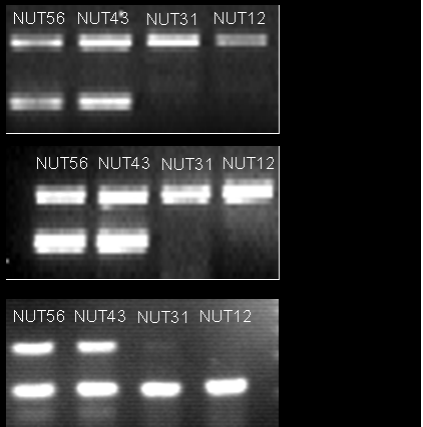
**Verification of gene expressions by semi-quantitative RT-PCR**. The RT-PCR analysis confirmed down-regulated expression of the identified genes in the endometrial tumors. The mRNA expressions of *Gpx3*, *Bgn *and *Tgfb3 *are shown here. NUT56 and NUT43 are specimens from normal/pre-malignant endometrium, whereas NUT31 and NUT12 are EAC specimens.

To confirm the microarray data, a semi quantitative RT-PCR was performed for all of the selected genes in ten of the cell lines included in the microarray experiment. The RT-PCR confirmed the gene expressions data from the microarray study in all measurements (Figure 3).

## Discussion

Gene expression profiling, in combination with statistical and classification methods, provides a powerful method of analyzing the complexity of cancer etiology. Genome-wide analysis of the expression patterns of neoplastic and non-/pre-malignant cells provides new information which can lead to identification of cellular pathways that might be affected by malignant transformation. In addition, global gene expression profiling facilitates the identification of new diagnostic markers and potential therapeutic targets.

In the present work, we have compared the expression patterns of EAC tumor cell lines with non-/pre-malignant endometrial samples. In total, 28 specimens were investigated, out of which 16 cell lines originated from EAC tumors and 12 from non-/pre-malignant lesions. Based on pathology, the tumors that developed in the N1 and F2 progenies were initially classified as EAC or other uterine tumors (Karlsson et al. 2007). However, in some cases, no malignant cells were detected in the removed cell mass when pathologically characterized. These tissue samples represent normal or pre-malignant endometrium, and hence provide new valuable information concerning the developmental stages during carcinogenesis. In a previous study, we re-classified the samples predicated on the expression patterns in combination with observed cell morphology/physiology. In the re-classification the expression of genes regulated by the TGF-β signaling pathway as well as global expression data were used, and two major groups with distinct differences in the expression patterns were recognized [9]. One group contained mostly samples earlier classified as EACs and only few earlier classified as non-/pre-malignant cell lines. This group was subsequently characterized as EACs, since the morphology and physiology of these cell lines (including those formerly classified as non-/pre-malignant), are in accordance with the typical properties of malignant cells in culture. The second group contained mostly samples previously classified as non-/pre-malignant, and a minority of EACs. This group was subsequently classified as non-/pre-malignant samples, since all cell lines in this group shared similar expression patterns, and displayed more or less normal morphological/physiological characteristics when cultured *in vitro*.

As expected, many genes showed differential expression between non-/pre-malignant endometrium and endometrial adenocarcinomas. In total, we found 354 genes that were significantly differentially expressed between the groups. In Figure 1, we present a clustering of the 50 genes with most significant differential expression between EACs and non-/pre-malignant samples. Generally, the genes that were found to be differentially expressed were down-regulated in the EAC group whereas the majority of the genes were more or less normally expressed in the non-/pre-malignant samples. The fact that the majority of the differentially expressed genes are down-regulated in the tumors implies that these genes are disruptive in EAC and may exhibit tumor suppressor features.

We also examined the gene functions of the top 50 genes using DAVID [10], were 31 genes were found to be involved in cellular processes known to be frequently disturbed in malignant cells (Figure 2a). Thus, the results from the gene function classification support the candidate gene selection from the significance analysis. The fact that several of these genes were involved in more than one process reflects the complexity of the cancer etiology (Figure 2b). These 31 genes could be considered a basis for an EAC signature.

When we performed the TDT analysis on genotyping data from the genome-wide screen with microsatellite markers, we could pin-point one gene, transforming growth factor beta 3, *Tgfb,3 *that was located in a susceptibility region identified in progenies from backcrosses with the BN background. Independent of tumor grade (malignant or non-/pre-malignant cell lines), we found a significant chi-square value from the TDT analysis (*p *< 0.05). Moreover, *Tgfb3 *was also found among the top 10 genes with differential expression between endometrial tumors and non-/pre-malignant endometrium. This result indicates that *Tgfb3 *is a susceptibility gene candidate and thus that the BDII strain might harbor an SNP involved in the initiation of EAC which will be further investigated. However, the expression data only correlated with grade of tumor as the gene was down-regulated in EAC and up-regulated in non-/pre-malignant cell lines regardless of the genetic background. An explanation for the differences might be that *Tgfb3 *expression is correlated to different genetic changes occurring during tumor development/progression. Since TGFβ3 is involved in multiple cellular processes the effects of genetic variation on mRNA expression may vary during tumor progression due to interactions with other affected genes. Thus, if the hypothesized genetic change in *Tgfb3 *is inherited, the effect on the expression level might be different in early and late stages of EAC.

To investigate whether more specific signature genes that differentiate between normal/pre-malignant endometrium and endometrial adenocarcinomas could be created, we used the classifier tool Weka, which contains a number of different machine learning algorithms. A majority of the algorithms applied performed very well in deriving classifiers that could classify the samples classes, with an average accuracy of 97.1%, and several algorithms performed at an accuracy of 100%. Depending on the type of classifier derived, it may or may not be possible to identify which individual gene was the most important for the classification result. For example, tree-based classifiers select a small subset of genes and place the most important of these at the root of the tree, whereas multi-layer perceptrons use all genes as input, and consequently the importance of each gene cannot be readily determined. In our results, 16 algorithms derived classifiers where the most important gene could be determined, and the two genes that were most frequently identified as top genes in the classifiers were *Gpx3 *(nine classifiers) and *Bgn *(seven classifiers). Based on the content of these classifiers, *Gpx3 *and *Bgn *are the best individual marker genes and should be investigated for their potential value in diagnosis of human endometrial adenocarcinoma. The value of *Gpx3 *and *Bgn *as signature genes is strengthened by the fact that both of them are within the 10 most significant genes in the microarray differential expression analysis described above.

Plasma glutathione peroxidase, *Gpx3*, exhibits a critical role in detoxifying reactive oxidative species and maintaining the genetic integrity of mammalian cells. *Gpx3 *has been found to be either deleted or highly methylated in exon 1 in prostate cancer cell lines [11,12] and in Barrett's tumorigenesis [13] and has been suggested to exhibit tumor suppressor activity. The tumor suppressor activity of *Gpx3 *is thought to be associated with its ability to repress the expression of *Met*. Expression data of *Met *in the present work implies that samples exhibiting an up-regulation of *Gpx3 *also show a low expression of *Met*. However, additional experimenting is essential to further evaluate the implications of loss of expression of *Gpx3 *in EAC carcinogenesis.

Gene expression of Biglycan, *Bgn*, is regulated by TGF-β signaling (dependent on functional smad2 signaling). We have previously shown down-regulation of several genes (several of these are among the top 50 genes presented herein) regulated by the TGF-β signaling pathway, indicating a disruptive TGF-β signaling. This result further strengthens our hypothesis of a dysfunctional TGF-β pathway in rat endometrial tumors. Furthermore, it has been shown that exogenously administered BGN induced pancreatic cancer cells to arrest in the G1 phase of the cell cycle, indicating a direct inhibiting effect on proliferation of BGN in cancerous cells [14].

In lung adenocarcinomas, the transcription of *Tgfb3 *has been suggested to be stimulated by collagen 1 through the PI3K/ERK pathway [15]. Our previous results have shown down-regulation of several pro-collagens [9] which might explain the decreased mRNA expression of *Tgfb3 *in our model. Interestingly, it has been shown that TGFB3 expression is up-regulated in human EAC and that TGFβ-3 exerts promoting effects on invasiveness [16], which is contradictive to our results. However, given the background that Van Themsche and co-workers used two commercial cell lines (i.e. KLE and HEC-1A) originating from elderly women (64 and 71, respectively), it seems that they represent the more invasive and non-estrogen dependent type II endometrial carcinoma, whereas our model represents estrogen-dependent endometrial carcinoma. This indicates that TGF-β signaling in the more aggressive type II endometrial carcinoma has tumor promoting effects, whereas it has been shown that in the estrogen-dependent type I endometrial carcinoma, the TGF-β signaling pathway is disrupted [17-19] and thus has a tumor inhibiting role. Further studies are needed to clarify the implications of down-regulated expression of *Tgfb*3 in rat EAC.

In conclusion, this work provides a unique data set of genes with distinct expression profiles that may serve as good candidates for further evaluations of genetic markers in EAC. Furthermore, by combining three different analysis approaches of the microarray data, we have identified a three-gene signature that seems to have underlying implications in EAC carcinogenesis. Table 2 summarizes the results from combining the three analysis methods for identifying a prominent EAC gene signature. As rat and human EAC carcinogenesis is very similar to each other, both proceeded by hyperplasias, the model can certainly be used for identifying potential markers and genes prior to human studies. Thus, we will perform detailed investigations of these three genes in the rat model which enables future evaluation in corresponding human tumors. Verification of the rat data in human EACs, and the use of comparative mapping data between human and rat will reveal the significance of using this signature for diagnostic and prognostic purposes in human uterine malignancies.

**Table 2:** Summary of results from Weka, gene function analysis and TDT analysis.

**Gene**	**Weka**	**Gene function analysis**	**TDT Analysis**
*Col1a1*		x (4)	
*Gpx3*	x	x (1)	
*Acta1*		x (3)	
*Serpine1*		x (3)	
*Tgfb3*		x (8)	x
*Wfdc1*		x (1)	
*Bgn*	x	x (1)	
*Tgfb1i1*		x (2)	
*Sparc*		x (1)	
*Lox*		x (2)	
*Thy1*		x (10)	
*Csrp1*		x (1)	
*Bnip3l*		x (5)	

*The numbers within the parentheses denote the frequency of cellular functions the gene is involved in.

## Materials and methods

### Animal crosses and tumor material

The animal material was derived from crosses between BDII/Han females and males from two non-susceptible rat strains, BN/Han and SPRD*Cu3*/Han, to produce an F1 progeny. Subsequently, an F2 offspring was produced by brother/sister mating of the F1 progeny and N1 progeny was produced by backcrossing the males to BDII females. The female progeny was palpated twice each week for identification of uterine tumors. Animals suspected to have tumors were euthanized and the tumor tissue surgically removed, subjected to pathological characterization and used to establish cell cultures (for details, see Helou et al. 2001). Here, we have investigated two groups of samples, i.e. cell lines established from tissues pathologically classified as endometrial tumors and cell lines established from tissues from normal/pre-malignant endometrium.

### Microarray experiments

The raw expression data of the microarray experiments were obtained from experiments described in Karlsson et al. 2007. Briefly, total RNA was extracted from harvested EAC and non/pre-malignant cell lines and labeled with Cy3 during cDNA synthesis. Universal rat reference RNA (Stratagene) was used as a common RNA reference (transcribed and labeled with Cy5) and thus hybridized to all arrays. We have employed the two-channel cDNA microarray format using the 18K (6000 clones in triplicates) rat 70 mer oligonucleotide arrays. The arrays were printed at the Swegene DNA microarray resource center in Lund, Sweden, by a BioRobotics MicroGrid 2 arrayer (Cambridge, UK) together with a split pin system. Each probe in the probe set (Rat 70 mer oligonucleotide set, ver 1.0, OPERON) was printed in triplicates at random positions on the arrays. The arrays were scanned in an Agilent Microarray scanner (Agilent technologies, USA). In total, 45 samples were arrayed. In the present work, we have focused the analysis on two groups of samples, constituting 29 arrays. These two groups represent early and more advanced stages of EAC and were therefore selected for the analyses in the present work, and is described more in detail in the discussion [9].

### Statistical analysis

The quality control of the GenePix Extraction results was performed using methods available from Bioconductor packages (marray, limma, arrayQuality) in the statistical software package R. Intensities of negative and positive controls were inspected, as well as spatial effects, M/A plots and signal-to-noise distributions. No unexpected results were found, thus indicating an overall high quality of hybridizations and scanning. Subsequent to image acquisition and analysis, the microarray data were up-loaded into the BioArray Software Environment (BASE) [20] for analysis. The data were cleaned to eliminate bad quality spots and subsequently print-tip lowess- and scale normalized. The replicates were merged during the normalization procedures. Spots that were present on less than 30 arrays (of the total 45) were rejected. The data was also subjected to variation filtering, i.e. all position-reporter pairs with standard deviation (SD) smaller than 0.8 were rejected. After cleaning, filtering and normalization procedures, 4336 probes/reporters out of 6000 remained. Significant differences in expression for reporters between the groups were assessed by applying Student's *t *test with a significance threshold of 0.05 and correction for multiple testing. Comparisons were made between EAC cell lines vs non/pre-malignant endometrial cell lines. The *p *values were adjusted using the False Discovery Rate (FDR) procedure [21].

In the classification analysis, fold change values extracted from the microarray data were imported into the Waikato environment for knowledge analysis (Weka, version 3.4.12) [22]. For each of the 28 samples, a "flag" (1 or 0) was set to signify group membership (cell lines from EAC tumors and non/pre-malignant lesions, respectively). The Weka software includes 70 different machine learning algorithms, each of which can be used to generate a classifier by learning from examples to distinguish between groups. We excluded all algorithms requiring discrete-valued input data, since these are not applicable to real-valued gene expression data sets. For each of the remaining 44 algorithms, the 28 samples were repeatedly divided into a training- and test set, using the leave-one-out cross validation method [23] and the average classification accuracy was recorded.

### Transmission disequilibrium test (TDT)

TDT statistics for the backcross progeny was performed on genotype data (Falck et al. manuscript in preparation) from microsatellite markers located adjacent to chromosomal regions harboring the identified classifiers and the top 50 genes with the most significant differential expression between the two groups. The TDT statistic for backcross progeny is defined as (*H*-*A*)^2^/(*H*+*A*), where *H *is the number of heterozygous animals and *A *is the number of animals homozygous for the BDII allele. The test has a χ^2 ^distribution with one degree of freedom. TDT statistics were calculated for markers adjacent to each gene in the EAC tumors versus non/pre-malignant lesions, and for differences between the two backgrounds, BDII/BN and BDII/SPRD, respectively.

### Gene function classification

A web-accessible program, the Database for Annotation, Visualization and Integrated Discovery, DAVID, was used to obtain an overview of the gene functions of the 50 genes with the highest differential expression between endometrial tumors and non-/pre-malignant endometrium. DAVID provides tools for functional annotation of genes and gene functional classification, in which large lists of genes can be rapidly reduced into functionally related groups of genes to help unravel the biological content. [10] We wanted to investigate whether these genes were involved in pathways/processes contributing to the cancer phenotype (increased proliferation, increased apoptosis etc) and therefore selected cellular processes typically involved in cancer development.

### Validation of the microarray data by semi-quantitative RT-PCR

Gene expressions for several genes associated with the TGF-beta pathway (*Col1a1*, *Col1a2*, *Col3a1*, *Col5a1, Ltbp2, Ctgf, Tgfb1i1*) were verified in a previous study [9]. In this study, gene expression changes detected in the microarray were validated on subset of tumor samples (n = 10) by semi-quantitative RT-PCR for *Tgfb3*, *Inhba*, *Wfdc1, Gpx3, Acta1, Serpine, Cpe, Bgn, Ssg1, Lox1, Bnip3I *and *Cnn*. Primer sets specific for these genes were constructed using the Primer3 program in the Center for Genome Research at the Whitehead Institute . The primers were designed to amplify across two or more different exons, thus ruling out DNA amplification. RT-PCR was performed on 500 ng of total RNA using Omniscript RT Kit according to the manufacturer's protocol (Qiagen, UK). Total 500 ng of total RNA was retro-transcribed to cDNA using 1× RT buffer, 2 mM dNTP mix, 1 μM Oligo-dT primer, 10 units RNA inhibitor, 4 units Omniscripts Reverse Transcriptase and RNAse free water for volume adjustment to 20 μl. PCR Amplification was performed in a 25 μl reaction mix using 10 μl of the RT reaction. The running conditions were as follow: 94°C for 2 min, 24–28 cycles of 30 sec at 94°C, 30 sec at 59°C, and 1 min at 72°C and followed by final extension of 72°C for 5 min.

## Competing interests

The authors declare that they have no competing interests.

## Authors' contributions

SK performed the cell culture experiments, microarray profiling laboratory work, conceived in its design and the statistical evaluations, the TDT analysis, participated in the Weka analysis, performed the RT-PCR validations of the microarray data and drafted the manuscript. BO performed the Weka analysis and helped to draft the manuscript. KKL was involved in the TDT analysis, the study design, performed the supervision of the study and helped in the manuscript preparation. All authors have read and approved the final version of the manuscript.

## Availability and requirements

The microarray data is available at: 

## Supplementary Material

Additional file 1 **The cross-validated average classification results from the Weka analysis**. 16 of the 49 algorithms derived classifiers could determine the most important gene for the classification, and the two genes that were most frequently identified as top genes in the classifiers were *Gpx3 *(nine classifiers) and *Bgn *(seven classifiers). Click here for file
