# Supplementary material for: Gene expression profiling predicts a three-gene expression signature of endometrial adenocarcinoma in a rat model
Source: Cancer Cell Int. 2009 May 8;9:12. doi: 10.1186/1475-2867-9-12 (PMC2687412; doi:10.1186/1475-2867-9-12)
Supplement: Additional file 1 — The cross-validated average classification results from the Weka analysis. 16 of the 49 algorithms derived classifiers could determine the most important gene for the classification, and the two genes that were most frequently identified as top genes in the classifiers were Gpx3 (nine classifiers) and Bgn (seven classifiers). [file 1475-2867-9-12-S1.doc]

| **Algorithm** | **Accuracy** | **Correct** | **Incorrect** | **EAC classed as Pre** | **Pre classed as EAC** | **Top gene** | **Erroneous EAC** | **Erroneous pre** |
| --- | --- | --- | --- | --- | --- | --- | --- | --- |
| ZeroR (baseline) | 59 | 17 | 12 | 0 | 12 |  |  |  |
| Ridor | 100 | 29 | 0 | 0 | 0 | Gpx3 |  |  |
| PART | 97 | 28 | 1 | 1 | 0 | Bgn | NUT81 |  |
| OneR | 100 | 29 | 0 | 0 | 0 | Gpx3 |  |  |
| Nnge | 100 | 29 | 0 | 0 | 0 | Many |  |  |
| Jrip | 97 | 28 | 1 | 0 | 1 | Gpx3 |  | NUT47 |
| DecisionTable | 100 | 29 | 0 | 0 | 0 | All |  |  |
| ConjunctiveRule | 100 | 29 | 0 | 0 | 0 | Gpx3 |  |  |
| REPTree | 97 | 28 | 1 | 0 | 1 | Bgn |  | NUT48 |
| RandomForest | 97 | 28 | 1 | 0 | 1 | Many |  | NUT82 |
| NBTree | 100 | 29 | 0 | 0 | 0 | Many |  |  |
| LMT | 100 | 29 | 0 | 0 | 0 | Many |  |  |
| J48 | 97 | 28 | 1 | 1 | 0 | Bgn | NUT81 |  |
| DecisionStump | 100 | 29 | 0 | 0 | 0 | Gpx3 |  |  |
| ADTree | 93 | 27 | 2 | 1 | 1 | Bgn | NUT81 | NUT82 |
| VF1 | 100 | 29 | 0 | 0 | 0 | All |  |  |
| HyperPipes | 97 | 28 | 1 | 1 | 0 | All | NUT81 |  |
| ThresholdSelector | 59 | 17 | 12 | 0 | 12 | All |  | All 12 |
| RandomCommittee | 100 | 29 | 0 | 0 | 0 | Many |  |  |
| OrdinalClassClassifier | 97 | 28 | 1 | 1 | 0 | Bgn | NUT81 |  |
| MultiClassClassifier | 100 | 29 | 0 | 0 | 0 | All |  |  |
| MultiBoostAB | 100 | 29 | 0 | 0 | 0 | Gpx3 |  |  |
| LogitBoost | 100 | 29 | 0 | 0 | 0 | Many |  |  |
| FilteredClassifier | 93 | 27 | 2 | 1 | 1 | Bgn | NUT81 | NUT82 |
| Decorate | 97 | 28 | 1 | 0 | 1 | Many |  | NUT82 |
| ClassificationViaRegression | 100 | 29 | 0 | 0 | 0 | Gpx3 |  |  |
| Bagging | 100 | 29 | 0 | 0 | 0 | Many |  |  |
| AttributeSelectedClassifier | 97 | 28 | 1 | 1 | 0 | Bgn | NUT81 |  |
| AdaBoostM1 | 100 | 29 | 0 | 0 | 0 | Gpx3 |  |  |
| LWL | 93 | 27 | 2 | 2 | 0 | All | RUT12, NUT39 |  |
| KStar | 86 | 25 | 4 | 0 | 4 | All |  | NUT48, NUT82,  NUT75, NUT43 |
| IBk | 100 | 29 | 0 | 0 | 0 | All |  |  |
| IB1 | 100 | 29 | 0 | 0 | 0 | All |  |  |
| VotedPerceptron | 97 | 28 | 1 | 0 | 1 | All |  | NUT48 |
| SMO | 100 | 29 | 0 | 0 | 0 | All |  |  |
| SimpleLogistic | 100 | 29 | 0 | 0 | 0 | Gpx3 |  |  |
| RBFNetwork | 100 | 29 | 0 | 0 | 0 | All |  |  |
| MultiLayerPerceptron | 100 | 29 | 0 | 0 | 0 | All |  |  |
| Logistic | 100 | 29 | 0 | 0 | 0 | All |  |  |
| NaiveBayesUpdatable | 100 | 29 | 0 | 0 | 0 | All |  |  |
| NaiveBayesSimple | 100 | 29 | 0 | 0 | 0 | All |  |  |
| NaiveBayes | 100 | 29 | 0 | 0 | 0 | All |  |  |
| BayesNet | 100 | 29 | 0 | 0 | 0 | All |  |  |

**Additional file 1 - The cross-validated average classification results from the Weka analysis.** 16 of the 49 algorithms derived classifiers could determine the most important gene for the classification, and the two genes that were most frequently identified as top genes in the classifiers were *Gpx3* (nine classifiers) and *Bgn* (seven classifiers).
